# Hepatocellular Carcinoma with Tumor Thrombus Extending from the Portal Vein to the Right Atrium

**DOI:** 10.7759/cureus.4689

**Published:** 2019-05-17

**Authors:** Laith Numan, Samia Asif, Omar K Abughanimeh

**Affiliations:** 1 Internal Medicine, University of Missouri-Kansas City School of Medicine, Kansas City, USA; 2 Hematology/Oncology, University of Nebraska Medical Center, Omaha, USA

**Keywords:** tumor thrombus, hepatocellular carcinoma, poor prognosis, esophageal variceal bleeding, liver cirrhosis

## Abstract

Hepatocellular carcinoma (HCC) is one of the most common malignant tumors. Tumor thrombus formation in advanced HCC stages is common and usually involves the hepatic or portal veins. The formation of tumor thrombus is considered a poor prognostic factor. Herein, we report a rare case wherein the thrombus extended to the inferior vena cava (IVC) reaching the right atrium without affecting the hemodynamic status. This is a 59-year-old male who presented with melena. He was found to have grade 3 esophageal varices with findings suggestive of recent bleeding associated with a large amount of blood in the gastric body that required banding. Computed tomography (CT) of the abdomen and pelvis showed multiple liver masses with an intraluminal IVC mass extending from the hepatic vein into the right atrium. A CT scan of the chest confirmed the presence of a tumor thrombus in the IVC extending to the right atrium. The patient declines surgical intervention and he was discharged. Unfortunately, he passed after a short period of time. In conclusion, tumor thrombus formation is common in HCC. However, expansion of the thrombus to IVC and right atrium is rare and indicates poor prognosis.

## Introduction

Hepatocellular carcinoma (HCC) is common, with an estimated annual incidence of 8.63 cases per 100,000 people in 2016 [1]. HCC is reported to be the fifth most common malignancy in the world [2]. According to the Surveillance, Epidemiology and End Results (SEER) Program, the observed five-year survival for HCC in 2011 was estimated at 20.35%, indicating high associated mortality rates. With the improvement in treatment mortalities, five-year survival has gradually improved over the 1975 to 2011 period [1]. Tumor thrombus (TT) formation is seen in advanced-stage HCC especially in the portal or hepatic veins. TT extension into the inferior vena cava (IVC) and subsequently into the right atrium (RA) is a rare entity and predictive of worse prognosis [3-4].

Literature review reveals prior cases of HCC with TT extending into RA with patients receiving treatment techniques ranging from simultaneous resection of liver tumor and TT, resection of TT alone, transcatheter arterial chemoembolization (TACE), transcatheter arterial embolization (TAE), sorafenib, and radiation therapy in multiple combinations. There appears to be no consensus on standardized mode of treatment. According to Wang et al., management of HCC with TT involving IVC/RA symptomatically, with TACE and surgically with hepatectomy and thrombectomy had a median survival of five, 4.5, and 19 months, respectively [5]. However, the surgical intervention itself carries a risk for postoperative complications, such as bile leakage for patients. Herein, we report a rare case of a patient with HCC with TT extending to IVC and RA without affecting the hemodynamic status. The choice of treatment modality can markedly affect patient outcomes, for that it remains essential to report each of these unusual cases so that we continue to identify the best course of management for these patients.

## Case presentation

A 59-year-old male presented with a history of untreated Hepatitis C and 50 pack-year smoking. He presented to the emergency department with black tarry loose bowel movements that started five days before the presentation. The melena was associated with cramping generalized abdominal pain and diarrhea. On review of systems, he complained of palpitation but denied any chest pain, shortness of breath, or lower limb swelling. He also mentioned that he noticed more than 20 pounds of weight loss in the last three months. Physical examination was remarkable for tachycardia with a regular rhythm, normal heart sounds, and no murmurs. On abdominal examination, he had epigastric tenderness on palpation, and he had non-tender hepatomegaly. Signs of liver cirrhosis were found on examination, such as spider angioma, gynecomastia, and mild palmar erythema.

**Figure 1:**
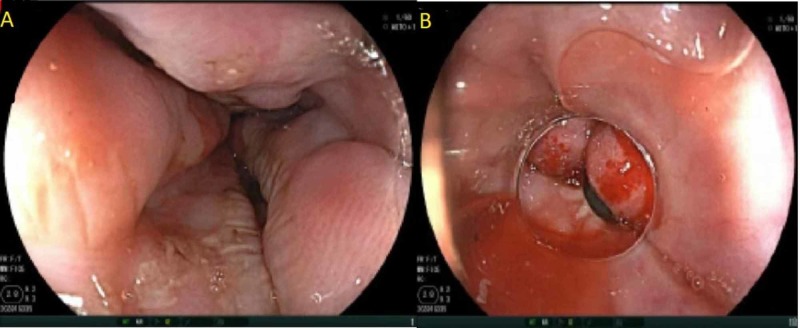
Endoscopic images of the esophageal varices. A: Grade 3 esophageal varices. B: Status post banding of the varices

Due to concerns of variceal bleeding, two large-bore intravenous catheters were inserted, a cross-match was done, and two units of packed red blood cell units were prepared. The patient was immediately started on octreotide and pantoprazole infusions with prophylactic ceftriaxone, and he was admitted directly to the intensive care unit. Gastroenterology team was consulted, and they performed an urgent esophagogastroduodenoscopy (EGD) that showed grade 3 esophageal varices with findings suggestive of recent bleeding associated with a large amount of blood in the gastric body that required banding (Figures 1A-1B). 

**Figure 2:**
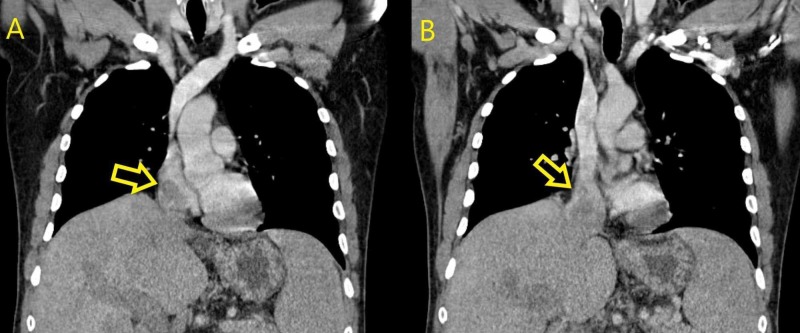
Coronal view of CT scan of the chest showing: A & B: Tumor thrombus making its way through the IVC to the right atrium CT, computed tomography; IVC, inferior vena cava

Upon further evaluation, the patient had a computed tomography (CT) of the abdomen and pelvis, which revealed multiple liver masses with an intraluminal IVC mass extending from the hepatic vein into the right atrium. This mass was thought to be a tumor thrombus that is extending up to the right atrium. Subsequently, a CT scan of the chest was done, and it confirmed the presence of a tumor thrombus in the IVC extending to the right atrium (Figures 2A-2B).

On further workup, his alpha-fetoprotein level was 53,320 ng/ml. Magnetic resonance imaging (MRI) of the abdomen confirmed the diagnosis of HCC. Oncology team evaluated the patient, and they determined that the patient was not a candidate for surgical or ablative therapies. The patient’s wishes were to avoid any invasive intervention for his thrombus and cancer. Therefore, he was discharged with a plan to receive palliative treatment with sorafenib as an outpatient. After two months of palliative treatment, the patient condition deteriorated, and he decided to switch to hospice, and he passed shortly after that.

## Discussion

HCC is an aggressive tumor with a tendency to grow into the blood vessels, resulting in tumor thrombus formation in major vessels like the portal vein or hepatic vein. TT is classified based on the anatomic location relative to the heart, which also determines the surgical approach if the decision is made to treat operatively. Type I or inferior hepatic TT involves IVC below the diaphragm; type II or superior hepatic TT involves IVC above the diaphragm but outside RA; and type III or intracardiac TT is TT above the diaphragm that reaches the RA [6]. IVC/RA TT is an infrequent though well-recognized occurrence with HCC, with a reported incidence of 3% to 4% of HCC patients who undergo diagnostic imaging [5,7-8]. It is believed that due to the lack of specific clinical signs, it may have been under-diagnosed, and the incidence is now increasing with the availability of improved imaging studies. IVC/RA TT poses an increased risk of pulmonary embolism, sudden cardiac arrest, and systemic metastases. Two cases, where IVC/RA TT resulted in acute Budd-Chiari syndrome, have been described as well [9].

Common clinical presentations of HCC with IVC/RA TT include abdominal pain and distension, mild fever, lower extremity edema and sometimes a palpable mass as well as the mere incidental discovery of TT. In some cases in the literature, patients presented with acute or subacute Budd-Chiari Syndrome as a result of IVC/RA TT as well as acute right heart failure and pulmonary embolism, manifesting as dyspnea with or without syncope [10]. IVC/RA TT may be diagnosed by Doppler ultrasound, CT scan, or MRI. In our case, the patient presented with variceal bleeding and the TT was discovered incidentally on imaging.

All patients with TT extending from retrohepatic IVC and RA are considered treatable by surgery if the primary hepatic tumor can be resected. Patients with decompensated liver cirrhosis, advanced stage of the primary tumor, and distant metastases are not considered surgical candidates and hence excluded from any benefit that can be rendered by surgical intervention. While Type I TT can be treated by radical hepatectomy and type II via abdominal approach and total hepatic vascular exclusion (THVE), once tumor extends fully into RA, cardiopulmonary bypass (extracorporeal circulation (ECC)) is essential during surgery [6,11]. In patients where TT enters only slightly into the RA, thrombectomy may still be performed under THVE without ECC. Postoperative adjuvant chemotherapy is required given a high likelihood of recurrence following surgery. Nonsurgical candidates can be offered TACE which may be repeated every one to two months. Sequential conformal radiotherapy (CRT) may be used with TACE in selected cases. Chang et al. reported three cases where oral thalidomide was used in the management of HCC with IVC/RA TT, resulting in regression of both intra-atrial TT as well as the primary tumor with improved symptoms and survival [10]. Li et al. reported the successful use of minimally invasive percutaneous microwave ablation (MWA) for treatment of HCC with IVC/RA TT in a 73-year-old gentleman who refused surgical intervention, without complication and followed up until 16 months, requiring repeated ablation procedures for tumor recurrence but who otherwise remained in good health [12]. This suggests MWA may be a treatment option for patients with advanced HCC with IVC/RA TT and patients such as the elderly who may not tolerate major surgery. Potential complications of MWA include pulmonary embolism from dislodgement of ablated TT and post-operative bilomas and biliary tract infection. Our patient was deemed a non-surgical candidate given his advanced cirrhosis and the variceal bleeding; in addition, the patient was against the idea of any invasive intervention and preferred to go the palliative route.

In a study by Wang et al., 56 patients with HCC and IVC/RA TT were evaluated over 10 years [5]. Overall, 25/56 patients underwent surgical treatment, 20/56 patients underwent TACE, and 11/56 patients opted for symptomatic management alone; one- and three-year survival rates for those managed with TACE or surgically with hepatectomy and thrombectomy as primary management were 15.0% and 5% versus 22.5% and 13.5%, respectively. For patients managed symptomatically alone, no one survived longer than one year. All patients treated surgically had complete removal of both primary tumor and TT; most were reported as having recurrences within the liver and lung metastases. Additional treatment options such as TACE, CRT, percutaneous microwave ablation and repeat hepatic resection were available to patients [5]. Our patient opted for symptomatic treatment alone, and unfortunately, he passed two months after the diagnosis.

A study by Treut et al. compared outcomes of patients undergoing major hepatectomy (resection of at least three contiguous Couinaud segments) with venous thrombectomy (VT) in 26 patients with 82 patients who underwent major hepatectomy alone [13]. This study concluded that median survival was four to five times shorter after major hepatectomy for HCC with TT than without and TT itself was a predictor of shorter survival [13]. Interestingly, prior studies have shown that if TT is removed alone, without hepatic tumor resection, there is some survival benefit as well [14-15]. While surgery to remove IVC/RA TT has been previously reported as technically challenging with postoperative deaths in earlier studies, recent literature as mentioned above, as well as in a study by Fukuda et al., showed postoperative complications were seen in few patients. This included pleural effusion, ascites, deep venous thrombosis, and heart failure but no hospital deaths were reported [16].

In brief, prior studies on the management of HCC with IVC/RA TT have demonstrated dismal prognosis in the absence of any treatment. Surgical intervention, despite its limitations such as whether a patient is a surgical candidate at all, and risks of the intervention itself such as intraoperative bleeding, has been shown to offer the best chances for improved survival. Multi-disciplinary treatments will be required post-operatively to prevent and control future HCC recurrences. Percutaneous ablation therapy is being explored as a treatment modality for elderly and advanced tumors. Further studies are needed to identify treatment strategies to allow improved mortality and morbidity in patients with HCC.

## Conclusions

Tumor thrombus formation is common in HCC. However, expansion of the thrombus to the right atrium is rare and indicates a poor prognosis, especially if left untreated. If the patient is a good surgical candidate, surgery carries the best chances. Other treatment modalities are being explored especially for elderly patients who are not surgical candidates.
